# Youth Codesign of a Mobile Phone App to Facilitate Self-Monitoring and Management of Mood Symptoms in Young People With Major Depression, Suicidal Ideation, and Self-Harm

**DOI:** 10.2196/mental.9041

**Published:** 2018-01-23

**Authors:** Sarah Elisabeth Hetrick, Jo Robinson, Eloise Burge, Ryan Blandon, Bianca Mobilio, Simon M Rice, Magenta B Simmons, Mario Alvarez-Jimenez, Simon Goodrich, Christopher G Davey

**Affiliations:** ^1^ Centre for Youth Mental Health University of Melbourne Melbourne Australia; ^2^ Department of Psychological Medicine University of Auckland Auckland New Zealand; ^3^ Orygen The National Centre of Excellence in Youth Mental Health Melbourne Australia; ^4^ Portable Web Innovation Company Melbourne Australia; ^5^ Royal Melbourne Institute of Technology University Melbourne Australia

**Keywords:** depression, suicidal ideation, suicide, attempted, self-injurious behavior, adolescent, young adult, cell phone

## Abstract

**Background:**

Effective treatment of depression in young people is critical, given its prevalence, impacts, and link to suicide. Clinical practice guidelines point to the need for regular monitoring of depression symptom severity and the emergence of suicidal ideation to track treatment progress and guide intervention delivery. Yet, this is seldom integrated in clinical practice.

**Objective:**

The objective of this study was to address the gap between guidelines about monitoring and real-world practice by codesigning an app with young people that allows for self-monitoring of mood and communication of this monitoring with a clinician.

**Methods:**

We engaged young people aged 18 to 25 years who had experienced depression, suicidal ideation including those who self-harm, as well as clinicians in a codesign process. We used a human-centered codesign *design studio* methodology where young people designed the features of the app first individually and then as a group. This resulted in a minimal viable product design, represented through low-fidelity hand-drawn wireframes. Clinicians were engaged throughout the process via focus groups.

**Results:**

The app incorporated a mood monitoring feature with innovative design aspects that allowed customization, and was named a “well-being tracker” in response to the need for a positive approach to this function. Brief personalized interventions designed to support young people in the intervals between face-to-face appointments were embedded in the app and were immediately available via pop-ups generated by a back-end algorithm within the well-being tracker. Issues regarding the safe incorporation of alerts generated by the app into face-to-face clinical services were raised by clinicians (ie, responding in a timely manner) and will need to be addressed during the full implementation of the app into clinical services.

**Conclusions:**

The potential to improve outcomes for young people via technology-based enhancement to interventions is enormous. Enhancing communication between young people and their clinicians about symptoms and treatment progress and increasing access to timely and evidence-based interventions are desirable outcomes. To achieve positive outcomes for young people using technology- (app) based interventions, it is critical to understand and incorporate, in a meaningful way, the expectations and motivations of both young people and clinicians.

## Introduction

Depressive disorders affect up to 25% of young people by the age of 18 years and account for the greatest global burden of disease in young people [1]. If untreated, there are high risks of developing further psychiatric disorders and impairments in occupational and social functioning [2,3]. Critically, depressive disorders confer a significant risk for suicidal ideation and suicide [4], the leading cause of death and disability in young people [5]. Early intervention is essential, and clinical practice guidelines recommend cognitive behavioral therapy or interpersonal therapy as first-line treatments, with fluoxetine as an adjunctive pharmacotherapeutic treatment when there is poor response to psychological treatment or when the depression is particularly severe and complex [6,7].

### Symptom Monitoring

Treatment recommendations within clinical practice guidelines are predicated on establishing the severity of depression symptoms and highlight the importance of monitoring depression symptom severity as a way to monitor treatment progress and inform ongoing treatment decision making. Specific depression symptom measurement tools have been suggested [6] to ensure the accurate reporting of symptoms. Moreover, clinicians are specifically instructed to monitor the emergence of suicidal ideation once a week for 4 weeks, and biweekly thereafter, upon commencing antidepressant medication, due to the known risks of this adverse outcome for young people taking antidepressants [6,8]. The accurate reporting of symptoms facilitates therapeutic monitoring [9] in terms of enhancing clinicians’ knowledge about the clients’ symptoms and risk, which can have a positive impact on treatment planning [10,11]. Young people have been shown to have faster symptom improvement when their clinicians receive feedback about treatment progress [12,13]. Furthermore, even without the clinician having this information, there is evidence that self-monitoring can improve outcomes across a range of health conditions [14-20].

However, there are significant challenges to implementing routine and meaningful monitoring [21-23]. Specific depression and suicidal ideation symptom measurement tools and regular timing of monitoring are often not incorporated into the processes and procedures in services; thus, monitoring is often not routine or meaningful. Time and resource constraints and relying on what can be irregular face-to-face client appointments (including clients who struggle to engage in treatment) also have an impact on the frequency and reliability of monitoring [21].

### Self-Monitoring

Self-monitoring offers a potential solution; this involves a young person regularly completing a symptom measurement tool in between their face-to-face appointments. This allows the capture of information about current depression and suicidal ideation symptoms. Self-monitoring is increasingly becoming part of self-management programs in general health areas [14-17] and has been shown to be effective in improving symptoms in adults with depression [24]. Self-monitoring overcomes problems with retrospective recall and captures the natural fluctuation in symptoms. It helps to overcome barriers to spontaneous reporting such as help-negation—a process of help avoidance or refusal [25-27]. Self-monitoring also serves as a feedback mechanism assisting individuals to notice fluctuations in their symptoms and how this might relate to changes in circumstances or life events. It can educate individuals about the conditions under which symptoms deteriorate or improve and thus can increase self-efficacy in relation to managing symptoms [20,28].

### Utilizing Technology

Web-based technology accessible via handheld devices offers the opportunity to implement real-time symptom self-monitoring. Such interventions have the potential to be more flexible, nonstigmatizing, accessible, and cost-effective [29] and to have significantly greater reach than traditional forms of treatment [30-34]. The use of technology in clinical practice has been shown to be acceptable to users and health professionals [35,36]. There is emerging evidence that young people may, in some circumstances, be more comfortable with technology over face-to-face intervention with therapists [37], and studies have shown that rates of disclosure of mental health difficulties and suicidal ideation are higher when paper and pencil or Web-based approaches are used relative to face-to-face assessment [9,10,38-42]. Similarly, young people are particularly enthusiastic users of smartphones and various mobile phone apps [43,44], and consider smartphones an acceptable form of support [45-47]. Technology thus has the potential to extend service delivery and improve treatment engagement and outcomes [9,48,49]. One study has developed monitoring within a self-directed online cognitive behavioral therapy (CBT) intervention (SPARX: Smart, Positive, Active, Realistic, X-Factor thoughts) [50] for young people with depression, showing this to be acceptable to users. We have developed and tested an online monitoring tool, delivered via a tablet, which was completed by young persons immediately before their face-to-face client appointment. Both young people and clinicians rated the tool favorably and found it useful for facilitating the timely exchange of information about symptoms and risks [10]. The results of the study indicated the potential for this intervention to be extended to allow symptom monitoring via a mobile phone app [10].

### Mobile Phone Apps

There are a large number of existing apps that monitor mood, although with a few notable exceptions [9,10,51,52], these have not been subject to research on their efficacy and safety, which has been highlighted as a major limitation of most apps that provide interventions for mental health issues [49,53]. Also emerging on the market are suicide prevention apps [54,55], particularly those that make Stanley and Brown’s safety planning intervention [56] available via an app [57,58]. A suite of recommendations are appearing with regard to the features that suicide prevention apps should include [57]. These include features such as immediate access to crisis helplines [59,60], a “hope box” [61], integrated safety planning that reminds people of their internal and external coping resources [57,59,62], and automated interventions [59]. Recommendations also include the ability to personalize the app, to pay attention to the potential impact of the colors used [60], and to ensure that the app is simple to use [57].

However, few apps to date have integrated self-monitoring of mood and suicidal ideation with brief interventions, including those recommended for suicide prevention apps, and apps that are designed to be integrated within clinical services have not been tested. Furthermore, few, if any, apps of this kind have been designed specifically with, and for, young people. We are aware of a very small body of research that has investigated the features young people would like to have incorporated in such an app [47,63], and of an app developed and beta-tested by Paul Stallard and colleagues in the United Kingdom in consultation with young people [55].

Consumer involvement in the concept development and design of apps for young people has been cited as crucial to ensure that app design better matches the needs and preferences of stakeholders [64], and participatory design is the epitome of this [65-67]. In this paper, we describe the codesign process and methodology and outcomes of designing a self-monitoring tool delivered via an app for young people receiving face-to-face clinical management of major depression.

## Methods

### Study Design

We used an overarching participatory design framework [66] and studio design methodology [68] to develop the app. This process goes beyond consultation and testing of already developed sketches or interventions; rather, we sought the active participation of users as codesigners throughout the design process [66]. This follows human-centered design principles, seeking to understand the needs of users and designing a solution with them to meet those needs. This approach recognizes the expertise of young people due to their age and, in this project, their lived experience of mental ill health. This expertise is valued just as much as the expertise of team members with formal qualifications (eg, researchers, clinicians). By using the principles of youth partnerships in research [69], the codesign process described below was undertaken within overarching complementary youth participation principles, including ensuring clear expectations from team members about the scope of each person’s contribution; being flexible, providing resources, and supporting involvement to limit barriers to participation; valuing diverse forms of experience (as previously described); involving more than one young person; reimbursing young people for their time; ensuring that all parties are benefiting from the experience (eg, skill development); avoiding tokenism to promote meaningful involvement; and providing feedback so that all team members are aware of what has been achieved.

### Setting and Participants

The study was undertaken in the Youth Mood Clinic (YMC) [70] at Orygen Youth Health (OYH); in a tertiary mental health service; and in secondary mental health services, headspace [71], all located in Melbourne, Australia. OYH is a public mental health service for young people aged 15-24 years. headspace services provide outpatient clinic-based assessment and individual face-to-face psychological and psychiatric treatment to young people aged 12-25 years [72]. Both OYH and headspace centers incorporate youth advisory groups, who facilitate youth participation in the design and delivery of services. In partnership with a digital design and technology company (Portable) and a youth participant who was a digital design student at a local university and being mentored by Portable, the study was undertaken with young people who were eligible to participate if they were aged 18 to 25 years and were current or former clients within these services for the treatment of depression (any level of severity), which may have also included suicidal ideation as well as self-harm (suicide-related behaviors). However, we excluded those who had experienced these suicide-related behaviors in the past 3 months.

Design for mental health technology must consider both young people and their clinicians [64]. All clinicians working within the YMC and headspace were eligible to participate.

### Participant Recruitment

Young people were informed of the study by their clinicians or by the coordinator of their youth advisory group. We also recruited via a chain-referral sampling method whereby participants informed young people they knew who might be eligible for the study. Those interested were directed to a Web page that contained information about the study and included an expression of interest form to complete. One of the investigators (SH), a clinical psychologist, then contacted those young people to further describe the study, ensure they met eligibility criteria, and provide details of the codesign workshop times and locations. Young people were able to attend as many or as few of the codesign workshops as they wanted to. All youth participants were reimbursed for their time (AU $30 per hour).

### App Design

On the basis of our previous study of an online monitoring tool, which was undertaken in the same clinical settings [10], and evidence that young people want technologies to enhance, not replace existing mental health services [73], it was proposed that the app would be designed to fit within and extend existing face-to-face services for young people with depression, including those at risk of suicide-related behaviors. On the basis of our previous study, it was proposed that the app would include the following:

- Onboarding (introduces the app, familiarizes the user with the purpose and functions of the app, and allows user registration)
- Monitoring of mood with feedback for young people and their clinicians
- An algorithm that generates automatic alerts at prespecified mood levels to encourage users to access help
- Prompts for the young person to complete a depression rating scale, the patient health questionnaire (PHQ-9) [74], and the 3-item suicide risk screener, which we developed in our pilot study [10], just before their appointment with their clinician.

### Codesign Workshops: Young People

The principal researcher (SH) and designers (EB, RB), along with the digital design student (BM), conducted 4 codesign workshops with young people and 2 focus groups with clinicians. Codesign workshops are designed to immerse participants and build a shared understanding of an issue to allow generation of concepts based on personal experience as well as previous research [66]. The methodology used was Design Studio [68], which is a key method within the user-centered agile design development field [75,76]. It involves the following: (1) young people individually sketching what they thought the app might look like and the features it should have; (2) young people presenting these sketches back to the group and gaining feedback on their design; (3) the group engaging in a team design using the best ideas (a process referred to as “feature prioritization”) from the individual design phase; (4) the “feature prioritization” from each workshop informing the following rounds of codesign; and (5) consolidating the best ideas into a final design that represents a minimal viable product design (a product developed with sufficient features for those involved in early testing), represented through wireframes.

### Codesign Workshops: Clinicians

One clinician workshop was held before the codesign workshops with young people, and one was held after the final codesign workshop. The initial clinician workshop was used to present the results of our online monitoring tool [10] and scope the needs and concerns of clinicians with regard to routine monitoring of their clients via an app. In the second workshop with clinicians, a young person who had participated in the codesign workshops presented the wireframes for the app. Feedback was sought on the design of the app as well as how it could be integrated with the face-to-face clinical services.

### Data Analysis

A general inductive approach was used for analysis, which allows findings to be derived in the context of focused objectives [77]. Thus, the analysis procedures have no specific label but are guided by the research objectives with the purpose of condensing the raw data. Although the objectives guide the analysis, findings are derived from analysis of the raw data rather than from a priori expectations driven by the objectives. The raw data were in the form of field notes from the focus groups and codesign workshops, as well as photographs of individual and team designs (photographs were not taken of participants to maintain anonymity). These raw data were summarized for the purposes of designing the app and highlighting the potential issues that need to be considered with regard to its use. The lead author as well as the app designers read the field notes and examined the photographs several times, and the lead author listened to audio recordings of the codesign workshops and focus groups, and a coding frame was developed via discussion. As new codes emerged, these were incorporated and field notes and photographs were re-examined. These codes were then organized into categories, which were conceptualized as themes. We were able to establish the trustworthiness of the data due to the iterative nature of the codesign workshops, and we sent the wireframes, as well as the results of the qualitative analysis, to young people and clinicians for comment.

### Risk Management

All young people were required to complete “a wellness plan” before participation, and robust procedures for ensuring the safety and well-being of participants were developed and implemented.

### Ethics Approval and Consent

Ethics approval was obtained from Melbourne Health Human Research Ethics Committee (Reference: HREC/15/MH/340; 2015.207), and written informed consent was gained from each clinician and young person.

## Results

### Codesign Workshops With Young People

A total of 8 young people attended at least 1 of the 4 codesign workshops: 5 attended 1 codesign workshop, 2 attended 2 workshops, 3 attended 3 workshops, and 1 young person attended all 4 workshops.

Of the participants, 3 identified as male and 8 as female. The mean age of participants was 21.4 years; the age of the youngest codesigner was 18 years and that of the oldest was 25 years.

Young people quickly developed a shared vision of what the app’s purpose was and of the key features it would include. They were enthusiastic about the idea of monitoring their mood: “Need some kind of pop-up—where you can note how you feel—are you in a good or bad place”; however, their overwhelming motivation was driven by the potential to develop something that would allow young people to access support in real time, when they needed it in between their face-to-face sessions, noting the limitations of current services, including telephone and online-based crisis support services: “If feeling really like [I] want to self-harm...need something that is immediately available.”

Young people were asked about key features that should be in the app. There was strong support for 5 key features that are described in detail below.

#### Onboarding

The onboarding process is the users’ first experience of an app and has to be designed so that it is easy to understand and use. Young people in the codesign workshops identified the importance of being able to customize the app. This included having the option to use the app as a guest or as a registered user. Young people highlighted the importance of being able to choose the welcome message and the color palate that was used in the app (highlighting that some colors might trigger negative mood states: “Childish colors...don't like brown...characters too bold...need something calm”) *.* Young people also discussed the need to customize the frequency and timing of mood monitoring and the distraction and care package activities that were available to them *.* They wanted to be able to choose who their support people were and customize a preprogramed default message that could be sent to a support person when they were very distressed or experiencing suicidal ideation. They also wanted to be able to customize the way that the mood monitoring ratings were calibrated and how mood monitoring feedback was presented (see below under Mood Monitoring).

Young people agreed that the onboarding process should be done with their clinician (“app should be opened up and started with the clinician”) and wanted the customization to allow the young person to modify and add new distraction and brief intervention activities as they learnt them: “make it an option to add to your list of things that help.”

#### Mood Monitoring

**Figure 1:**
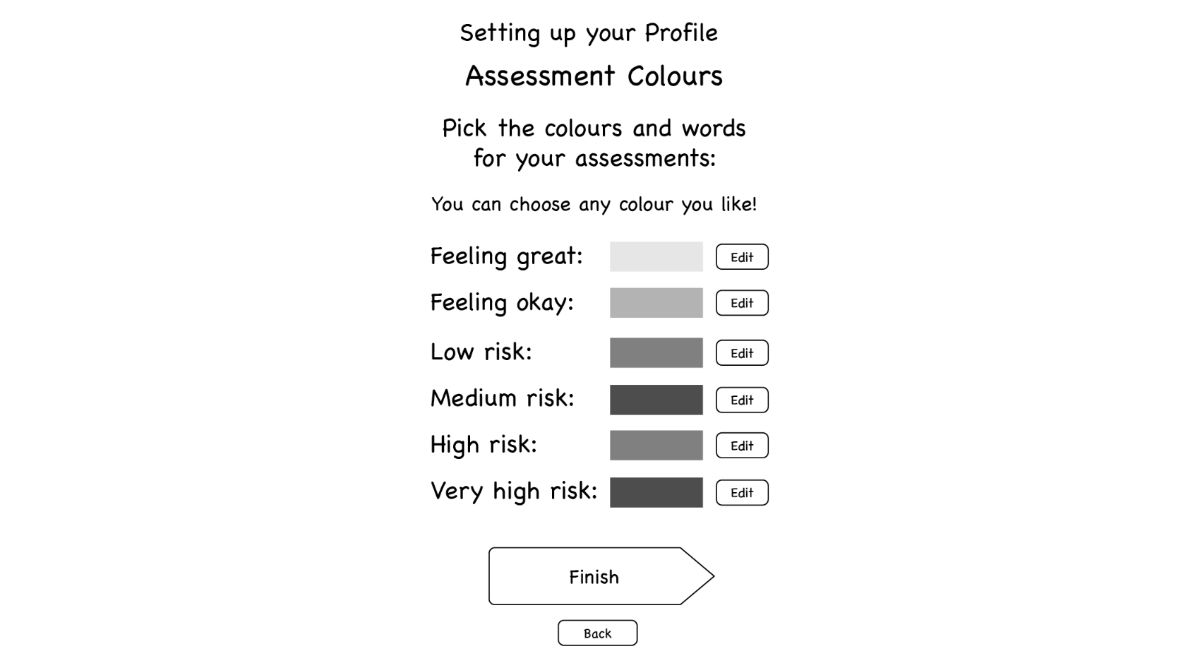
Customization of mood monitoring feature (well-being check) using colors.

**Figure 2:**
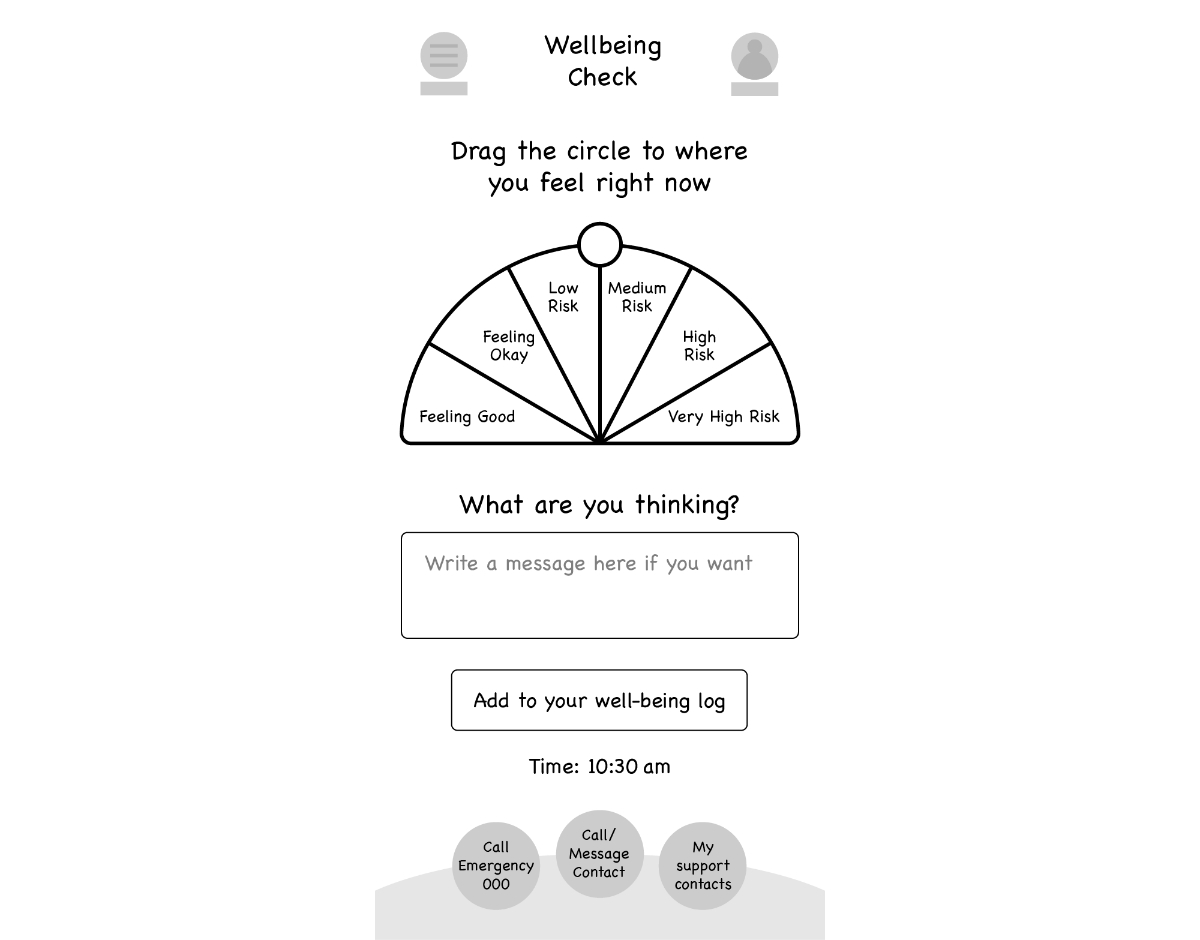
Well-being check.

Young people wanted the app to include a feature to monitor their mood. They highlighted that generic descriptors of mood and generic numerical ratings of mood were not necessarily useful or relevant across the population of young people who might use the app. They were clear that the app should not over simplify mood states, instead preferring a more nuanced monitoring approach: “I don’t like sad, happy or in-between faces...there’s so much more to emotions.” Thus, a key innovation for this mood monitoring feature, and again in line with recommendations about customization [60], was to establish both a preferred color system for mood that for each young person equated with their own customized ratings and, with involvement of their clinician, “trigger points”’ for high distress, and possible onset of suicide-related behaviors: “realized for me a 9 might be like super high...for someone else 6 might be really high...or I might consistently press a 4 every day and that is ok for me...so we’ve sort of made it customizable” (see Figures 1 and 2).

Young people described the importance of having a feature where they could enter potential influences on their mood: “space to write comments about what happened that day...you can record your thoughts.” However, they were clear that the annotations about what had impacted their negative mood should not be automatically displayed but go in a separate section that they could open if they wanted to, stating that it might not be helpful to dwell on this if something negative had happened. This highlighted again that the app should ensure a positive approach to monitoring, allowing rating of positive mood states with a potential further innovation being to provide notifications about what appears to improve mood “I don’t want to see an unhappy face on the calendar everyday” or “don’t use the word crisis.” Thus, this feature became known as a “well-being check.” They were also clear that various approaches to displaying the mood “ratings” over time should be provided so that young people would have choice about how they saw this (eg, a graph or calendar displaying colors over time).

Finally, young people designed an algorithm that was built into the mood monitoring feature that linked, via a pop-up, to appropriate levels of intervention (described below) corresponding to their mood state including for “trigger points” that indicate moderate and high levels of distress, which is individualized and quantified according to the young person’s own criteria. Table 1 describes a potential algorithm that is subject to further testing and validation.

#### Distraction

Young people were unanimous in wanting real-time distractions that provided an immersive experience and diversion from intense and distressing mood states, including suicidal ideation. As stated above, they highlighted the need to ensure customization of these distraction interventions to suit the varying needs of different young people. The types of distractions young people discussed as being useful included the following: meditation with simple tips given to calm the user, games, music, breathing exercises, and videos (eg, inspirational, funny, or of their support people providing support messages).

This feature represents the “internal coping strategies” element of the Stanley and Brown’s safety planning app [56]. Distraction is also a core intervention in dialectical behavioral therapy (DBT) [78], an evidence-based intervention for young people engaging in self-harm [79].

#### Brief Interventions

These brief interventions were designed by young people to be something that could be used regularly, rather than only when they were experiencing high distress. They could include all of the distraction interventions described above and also include features such as a photo album that contained meaningful photos, or photos that induced a positive emotion, supportive messages from friends and loved ones and messages that induced positive emotions, links to music playlists (eg, Spotify), and inspirational quotes (with the option of quotes generated by the user or app generated).

This feature is consistent with the interventions designed to increase distress tolerance, such as DBT and CBT, both evidence-based interventions for young people engaging in self-harm [78-80]. It is also reminiscent of the concept of a “hope box,” which is a technique used in DBT and CBT that has been recommended for inclusion in apps that have a suicide prevention function [61].

**Table 1:** Algorithm of “interventions” made available according to mood monitor ratings.

Well-being rating	Levels of intervention
Low risk	Positive affirmation
Low to medium risk	Positive affirmation
	Link to care package
Medium risk	Positive affirmation
	Link to care package
Medium to high risk	Link to distraction
	Prompt to ask if user wants to talk to a friend or support person
	Prompt to ask if user wants to fix an appointment with a clinician
High risk	*If user responds to the prompt stating they feel safe:*
	Prompt to ask if user wants to fix an appointment with a clinician
	Prompt them to schedule another self-assessment
	Link to distraction
	*If user responds to the prompt stating they are unsure if safe:*
	Prompt to ask if user needs support line: if yes, preprogramed text message to support person and 24-hour crisis support line number selected; clinician notified that user has accessed support. If no, user prompted to fix an appointment with a clinician and schedule another self-assessment
	Link to distraction

#### Safety Features

In line with recommendations [59,60], young people agreed that it was important that on every screen of the app there was a one-touch option to access emergency services for immediate crisis intervention, as well as a one-touch option to call or message (using a preprogramed default message) their support person, and a one-touch option to see their list of support contact people. Young people provided a compelling rationale for this one-touch option that allows a preprogramed default message to be sent to their support person. They clearly stated that it was very difficult to both scroll through contact lists to find a phone number and to construct a message about their distress and need for help in times when they were experiencing intense levels of distress and suicidal ideation: “even just like if you just preprogrammed into the app just you know like a list of people you care about, care about you, people you feel safe with...it comes up as an option”; “it will come up with ‘do you need to call a family member or friend or selected member’ who knows you are using this app and knows your history.”

Young people showed insight into the constraints of the services in which they were engaged. They were aware of the working hours of clinicians and did not expect clinicians to respond to distress messages after their working hours. They were keenly aware that crisis services, such as telephone helplines, were not always able to provide a timely service. Young people did think it was important that their clinician had access to the information generated from their mood monitoring in their face-to-face sessions, including when they had accessed the distraction function, contacted their support person, or used emergency or crisis services:

The idea isn’t that you have access to your clinicians at all times of day...it is very clear that this is not what this is...the idea is that it is to be a log so you and your clinician can see over the past month what has been good, what has been bad, what has been happening...

### Clinician Workshops

In total, 16 clinicians participated in the workshops. These clinicians were from a range of professional backgrounds including clinical psychologists, psychiatrists, social workers, and occupational therapists. They all had considerable experience working with young people with severe mood disorders. Many of them worked across both the YMC and a headspace clinical service.

#### Clinician Responsibility in Responding to Mood Monitoring

Clinicians at both workshops expressed concerns regarding the clinical responsibilities that would ensue after being informed by the app about high levels of distress and suicidal ideation. They articulated that once this information was known, action would need to be taken to mitigate the risk of suicide. They were uncomfortable about receiving these notifications when they were not present in their clinical roles (ie, in the evenings, on weekends, and while they were on leave). Clinicians expressed concerns that developing a mechanism for being notified of a young person’s distress and responding to this would require significant additional clinical resourcing.

#### Managing Expectations

Clinicians highlighted the importance of clarifying and managing the expectations of young people using the app. Unmet expectations, for example, if a young person did not receive an immediate response from a support person, has the potential to result in unintended harm. Suggestions to manage this included ensuring that clinicians had choice over which clients they would use the app with and working closely with the young people in the onboarding stage to familiarize the young person with the app. They highlighted how important it was to ensure that young people were clear about when and what sort of response they should expect from their clinician. In this regard, they highlighted that receiving emails documenting the mood monitoring results for a client would not be appropriate because it was not possible to guarantee a timely response due to workload or circumstances such as the clinician being on leave. The preference of clinicians was to view the mood monitoring information only in their face-to-face sessions with young people. They emphasized that they wanted knowledge of any suicidal crisis and related intervention via traditional means such as notification from other clinicians, as well as from the information within the app that they would view during the face-to-face session with their client. They did note that it would be clinically useful to incorporate a discussion of the mood monitoring results and the potential influences on mood states, as well as what and how young people had used the distraction and care package interventions into their face-to-face clinical sessions. They also suggested that it might be useful to ensure a printable version of the mood monitoring results, for example, a PDF document that could be used in sessions and included in the clinical record. It was the clinician’s view that, although the app allowed the young person to monitor his or her mood in real time, it would not be feasible for clinicians to have access to or respond to these data in real time. They suggested that the app should be considered as, and more appropriately named, a “digital diary” rather than “mood monitoring,” a term that gives the impression that it is a live monitoring device.

The clinician interface tool that was designed as part of the app was designed to be utilized as a separate Web app tool through which clinicians could view the young people under their care and their details. In this tool, clinicians would be able to view the young person’s mood through a calendar or chart function to visually see details of mood ratings over time. Clinicians would also be able to see if any emergency calls were made, what interventions (either from the care packages or distractions) were utilized, and their impact on mood ratings. Allowing the clinician to see this information in the clinician's own time allows them to access the information quickly and alleviates the perception that a young person could rely on the digital diary as a source of immediate or on-demand treatment.

With regard to managing the expectations of young people, they also made the point that young people also needed to be made aware that their support people may not always be available, for example, if there was a delay in the message being sent due to network problems, if their support person’s phone was not charged, if they were away from their phone, or if they were unwell. Clinicians raised some concerns at both workshops about the potential burden on the support person and how this person needed to be carefully selected and made aware that they were going to be contacted by the young person in this way.

#### Potential Adverse Therapeutic Effects of the App

Clinicians highlighted a potentially counterproductive effect of one feature of the app (the function allowing a preprogramed default message to be sent when a young person was distressed), in that it could create a learned helplessness with regard to help seeking. Clinicians were concerned that young people might develop an expectation that others should respond and initiate supportive contact when they were in distress, discouraging the young person to learn active help-seeking skills.

## Discussion

### Principal Findings

The development of this app utilized a codesign process, predominantly with young people but also involving clinicians. Young people and clinicians were enthusiastic about the app including mood monitoring as a key function. Young people wanted a positive approach to this and developed a creative and innovative approach to this function that allowed an individual to customize their well-being scale to indicate when and what type of intervention was needed [50]. The provision of real-time support as well as issues with the accessibility to timely response in times of crisis were considered critical. One way of addressing this was to design embedded interventions to enable young people to manage their own distress. The development of the app, using a codesign process, is a significant advance on many of the currently available apps (and other technology-based interventions) that have been designed to address mental ill health because of the incorporation of the youth perspective. The inclusion of both clinicians and young people in a codesign process highlighted disparate needs, motivations, and intentions for the app, and by incorporating the views of both, the app has promise as a tool to assist both clinicians and young people in the management of depression and suicide-related behaviors.

### Limitations

The codesign process used in this study has been undertaken with help-seeking young people who were predominantly recruited via their clinicians or the coordinator of the youth advisory group in which they were involved. To ensure we could safely manage risk, we imposed criteria on inclusion that required that we only include older adolescents and young adults who had not experienced suicide-related behaviors within the previous 3 months. We acknowledge that some young people may have been unwilling to participate given their participation in the codesign workshops meant others would be aware of their history of depression and suicide-related behaviors. Thus, the app may not be relevant or acceptable to all young people or to those accessing different kinds of services. Young people in the codesign workshops had some awareness of this and highlighted the need for customization because “young people” are not a homogenous group, but all have different needs and preferences. Customization has been highlighted as important by adolescents in similar codesign processes [50]. It is important to note that the app has not been designed for young people who are not engaged in face-to-face treatment.

Although our app conforms to clinical practice guideline recommendations with regard to routine monitoring of symptoms (depression) and medication side effects (suicidal ideation) and its prototype has been beta-tested [10], there is a need to robustly test the app for efficacy and safety, including testing that the innovative mood rating function is a reliable and valid measure of mood compared with validated measures such as the PHQ-9 and our 3-item suicide risk screener [10].

Through the process of codesign, we have been made aware of some of the barriers to implementing this app into face-to-face clinical care. Further work will be required to tailor implementation of the app into various service settings and governance structures. This is potentially challenging, given the different processes used across various services, and highlights that health providers and young people have different expectations and preferences with regard to the use of technology in mental health care.

### Comparison With Prior Study

The functionality of the app is consistent with prototypes developed for adults experiencing depression [9,51,52], with recommendations for suicide and self-harm prevention apps [57,59-62], with what young people in other studies of app development have described as potentially useful [47,63], and with a recently developed app developed in the United Kingdom for young people at risk of self-harm [55]. That young people and clinicians wanted mood monitoring as a key function is consistent with the previous finding that mood monitoring is an acceptable and safe function for users [9,10]. Young people highlighted the need for a positive approach to mood monitoring, which also aligns with previous findings [65,51], and resulted in this function being named a “well-being tracker.”

The inclusion of distractions, which are essentially self-soothing interventions or “internal coping strategies” as well as one-touch access to personal support, is consistent with Stanley and Brown’s widely used safety planning intervention [56]. These interventions are also consistent with emotional regulation strategies used in DBT, an intervention with evidence of its efficacy in reducing self-harm [79,81,82]. Overall, the key features that young people wanted to include in the app are consistent with the recommendations for apps in the field of suicide prevention and are evidence based, thus addressing concerns raised in the literature about the types of nonevidence-based interventions that are provided in many commercially available apps [49].

Young people clearly expressed a need to have mechanisms to overcome help-seeking barriers when they were very distressed, consistent with what is described as help-negation in the literature [25]. This echoes findings relevant to the development of monitoring in the context of the SPARX intervention [50]. Young people in our study developed an innovative approach whereby preprogramed messages could be developed for automated delivery to the young person’s support person. However, clinicians were concerned that this function could be counter-therapeutic in terms of preventing young people taking an active role in their help seeking. This highlights the need to ensure that innovation is undertaken in the context of high-quality evaluation to ensure that there are no unintended adverse therapeutic impacts [50,65] and to document whether these clinical concerns are founded.

Clinicians also raised significant concerns about the implications of real-time mood monitoring with regard to ensuring the safety of their clients, consistent with concerns raised in similar studies [50]. They highlighted that there was no capacity for clinicians to receive or respond to mood monitoring data from their clients, except within their scheduled face-to-face client appointments, where both young people and clinicians agreed the information was potentially clinically useful. Our previous study and results from the development of monitoring for SPARX highlight the potential for monitoring to enhance communication between young people and clinicians and engagement in treatment [10,50].

A key issue is balancing the needs of young people with the potential clinical burden, and the need for moderation in a landscape where mental health services are already underfunded and overstretched. Although in Australia the integration of technology into clinical care is a national priority [83], the additional resourcing to ensure this is done in a safe and effective way has not followed this policy direction. Greater resourcing of mental health services would, for example, ensure that the full advantage of real-time mood monitoring could be realized in terms of identifying and intervening with young people in real time to deal with acute risk of suicide rather than using the app as a digital diary.

### Conclusions

Extending and enhancing treatment services for young people with depression are important goals given the necessity to ensure that early and evidence-based interventions are delivered to this group. There is significant potential to improve the lives of young people experiencing depression via the provision of technology-based enhancement to interventions, although it may mean significant redesign of current mental health service systems. Our findings have highlighted the critical need to ensure both clinicians and young people are involved in the development of these kinds of interventions to ensure the needs of young people as well as clinicians, and the services in which they are working, are addressed in the design of the intervention.

For clinicians, the app has been designed to assist with ensuring guideline concordant care (symptom monitoring). For young people, it addresses their need for support when they experience distress and suicidal ideation in between their face-to-face appointments. Thus, the app has the potential to enhance communication between young people and their clinicians about symptoms and treatment progress and increase access to timely and evidence-based interventions. Understanding and incorporating the expectations and motivations of both young people and their clinicians are critical to ensure that this can be done.

## Abbreviations

CBT: cognitive behavioral therapy
DBT: dialectical behavioral therapy
OYH: Orygen Youth Health
PHQ: patient health questionnaire
SPARX: Smart, Positive, Active, Realistic, X-Factor thoughts
YMC: Youth Mood Clinic
